# Extension osteotomy of the metacarpal I and ligamentoplasty of the trapeziometacarpal joint for the treatment of early-stage osteoarthritis and instability of the trapeziometacarpal joint

**DOI:** 10.1007/s00402-023-04883-1

**Published:** 2023-05-19

**Authors:** Philipp Honigmann, Marco Keller, Noémie Devaux-Voumard, Enrico Coppo, Damian Sutter

**Affiliations:** 1 Hand and Peripheral Nerve Surgery, Department of Orthopaedic Surgery and Traumatology, Kantonsspital Baselland (Bruderholz, Liestal, Laufen), Bruderholz, Switzerland; 2 Medical Additive Manufacturing Research Group (MAM), Department of Biomedical Engineering, University of Basel, Allschwil, Switzerland; 3 Amsterdam UMC, Department of Biomedical Engineering and Physics, University of Amsterdam, Amsterdam Movement Sciences, Amsterdam, The Netherlands; 4 Division of Plastic Surgery and Hand Surgery, University Hospital Zürich, University of Zürich, Zurich, Switzerland; 5 Department of Plastic and Hand Surgery, Inselspital, University Hospital, University of Bern, Bern, Switzerland

**Keywords:** (Wilson) extension osteotomy, Trapeziometacarpal joint, First carpometacarpal joint, Saddle joint, Ligamentoplasty, Osteoarthritis

## Abstract

Osteoarthritis (OA) is a common disease of the first carpo-metacarpal (CMC I) joint. Biomechanical factors promoting OA are the shape of the CMC I-joint, being a biconcave-convex saddle joint with high mobility and the increased instability caused by joint space narrowing, ligamentous laxity, and direction of force transmission of the abductor pollicis longus (APL) tendon during adduction. The closing wedge osteotomy of the base of the first metacarpal is joint preserving treatment option. We combine this closing wedge osteotomy with a ligamentoplasty to stabilize the joint. In this manuscript, we provide a detailed description of the indication, discuss biomechanical aspects and the surgical technique in detail.

## Introduction

Osteoarthritis is a common disease of the first carpo-metacarpal (CMC I) joint. The shape of the CMC I-joint, which is a biconcave-convex saddle joint, allows for increased mobility. Instability is caused by joint space narrowing, increasing insufficiency of the CMC I-joint ligaments and the direction of force transmission of the APL-tendon during adduction of the first metacarpal bone [1, 2]. Instability of the joint and incorrect axial load distribution contributes to the formation of osteoarthritis. Joint compression (contact) forces are high. One kilogram of applied force during a simple pinch grip leads to three kilograms of transmitted force at the interphalangeal joint, 5.4 kg at the metacarpophalangeal joint, and 12.0 kg at the carpometacarpal joint [3].

A ligament reconstruction using a flexor carpi radialis (FCR) tendon slip has been proposed as treatment option in pre-arthritic as well as in higher grade saddle-joint osteoarthritis by Eaton and confirmed by long term follow-up results [4, 5]. Of the 38 cases with a minimum follow-up of 4 and an average follow-up of 7 years, 8 were stage I, 11 stage II, 10 stage III and 9 stage IV. Excellent results were achieved in 88% of the patients with stage I, 55% with stage II and IV and in 60% with stage III. In another series of 38 patients published by Lane and Henly, 35 cases (67%) had excellent results, 11 (30%) had good results and 1 (3%) had a poor result [6]. To neutralize the two main acting forces on the first metacarpal, the adductor pollicis (AP) and the abductor pollicis longus (APL), Brunelli used a distally based APL tendon-slip which is pulled through the base of the first and second metacarpal bone and sutured to the periosteum and fascia [2]. Various techniques and their modifications are summarized in a publication of operative techniques by Langer et al. [7].

The surgical treatment of early stage CMC I osteoarthritis performing an extension osteotomy at the base of the first metacarpal has first been described in 1973 by J.N. Wilson [8]. Biomechanical studies have shown the extension osteotomy to shift load transfer to the less commonly affected dorsal joint aspect and reduce joint laxity [9, 10]. Various authors described this technique using box- and/or k-wiring with or without stabilization of the joint using a tendon slip from various origins like FCR [11]. Also, minimally invasive procedures were described drilling “blindly” from the first into the second metacarpal bone followed by a mini tight rope suspension [12].

In 2001, Tomaino raised the question of ligament reconstruction or thumb metacarpal extension osteotomy being more beneficial to treat osteoarthritis of the CMC I joint [13]. More than 20 years later, Chiang et al. found that osteotomy alone without ligamentous reconstruction may be inadequate to provide the stabilization required for manual abilities like grasping and pinching, particularly in the long-term [11].

We believe that both procedures should be combined, the closing-wedge extension osteotomy of the first metacarpal bone to correct the load axis and a ligamentoplasty to sufficiently stabilize the CMC I–joint. In contrast to osteotomy fixation with K-wires, staples or osteosutures, we aim for primary stability which allows for early postoperative active motion. Therefore, we use a dorsally applied T-shaped plate combined with a modified Brunelli-ligamentoplasty using an APL-tendon slip for early-stage CMC I-joint osteoarthritis to align the load axis, stabilize the CMC I-joint and hence prevent osteoarthritis progression.

## Anatomy

Branches of the superficial radial nerve need to be identified and preserved. The radial artery passes from proximal radial into the first webspace below the interval of the extensor pollicis longus tendon and the APL and Extensor pollicis brevis tendon. The here described approach allows sufficient visualization of the base of the metacarpal I and II and the trapezium as well as the muscles of the first web space.

## Indications/contraindications

The mere morphologic severity of CMC I-osteoarthritis according to the radiological classification of Eaton and Littler is insufficient to describe the correct indication for surgery [4]. Anamnestic and clinical features outweigh the radiological degenerative signs. The important elements to consider during clinical and radiological assessment are the trapeziometacarpal joint arthritis (TMA), trapeziometacarpal instability/subluxation (TMI) and the condition of the scaphotrapezoid joint (STT) [1]. According to the classification of Allieu, a TMA resulting in narrowing of around 50% (TMA stage 1 and 2), a reducible subluxation of more than 1/3 of the joint’s surface area of the first metacarpal base (TMI stage 3) and anatomical alteration or decrease of half or less of the STT-joint space (STT stage 1) are the limits for indication of our described procedure [1] (Table 1).

**Table 1:** Level of arthritis and joint instability of the trapeziometacarpal joint as well as the level of arthritis of the scaphotrapeziotrapezoidal joint according to Allieu et al.

Stage	Trapeziometacarpal joint arthritis (TMS)	Trapeziometacarpal joint instability (TMS)	Alteration of scaphotrapezoidal joint (STT)
0	No jo int narrowing (painful and unstable joint)	Reducible subluxation,painful and unstable joint	Thumb basa ljoint arthritis without alteration of SIT
1	Start of narrowing (< 50%)	Reducible subluxation but with incomplete reintegration	Anatom ical alteration or decrease half or less of the joint space
2	Marked narrowing (> 50%)	Non-reducible subluxation,less than 1/3 of the jo int’s area at the first metacarpal base	Joint space is scarcely visible
3	Disappea rance of joi nt space, bone erosion	Subluxat ion over 1/3 of the joint's area at the first metacarpal base	Presence of erosion,sclerosis, and irregularities

## Technique

### Biomechanical considerations and preoperative planning

Wilson proposed a 20° extension osteotomy [8]. He did not calculate exact values according to the individual differences of the anatomical axis (AA) and mechanical axis (MA) of the MC I. Whereas the anatomical axis is the inertia axis of the bone, the mechanical axis is calculated according to the following:

A circle is projected over the MC I head. The centre of this circle is the distal final point of the mechanical axis. This technique is based on the method described in the International Society of Biomechanics (ISB), although it also considers the three-dimensional orientation of the bone [14].

**Fig. 1:**
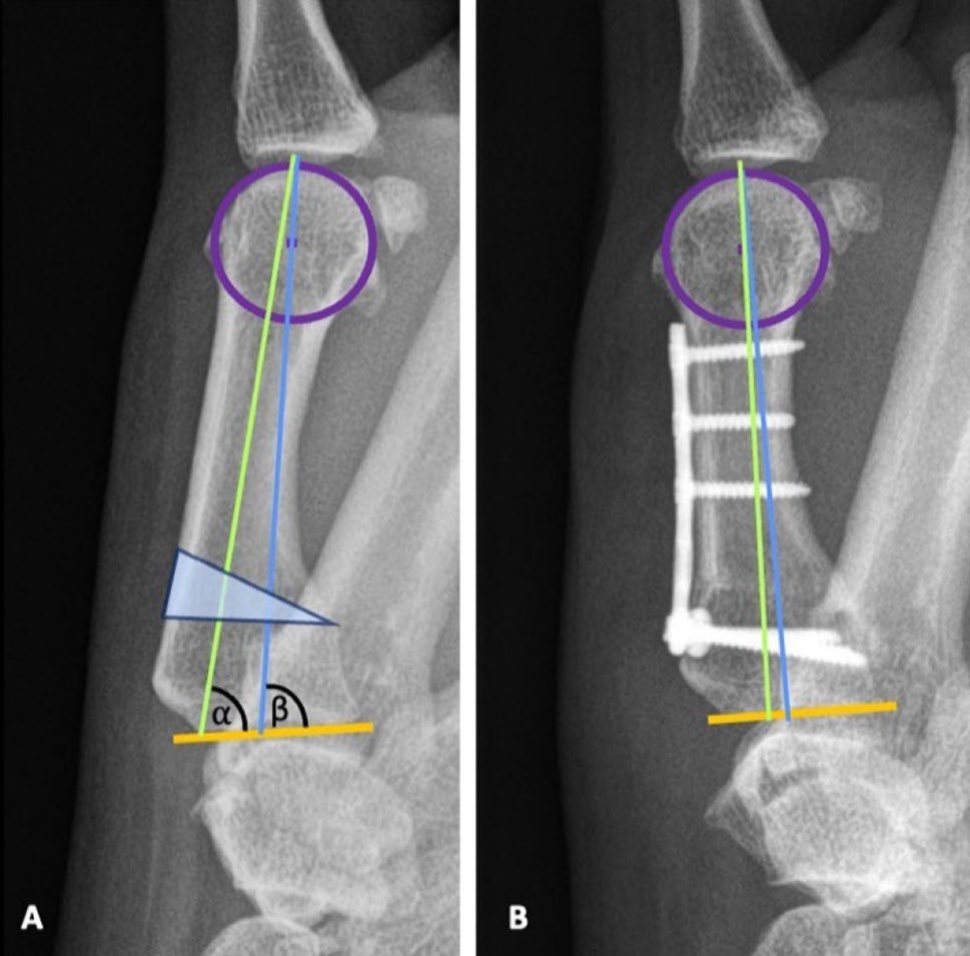
Visualisation of the correction of the axes from the pre- (**A**) to the postoperative (**B**) state (circle in the MC I head (purple), anatomical Axis (green), mechanical axis (blue), proximal joint line—PJL (orange), angle between PJL and AA (Alpha), angle between PJL and MA (Beta), osteotomy wedge (light blue))

**Fig. 2:**
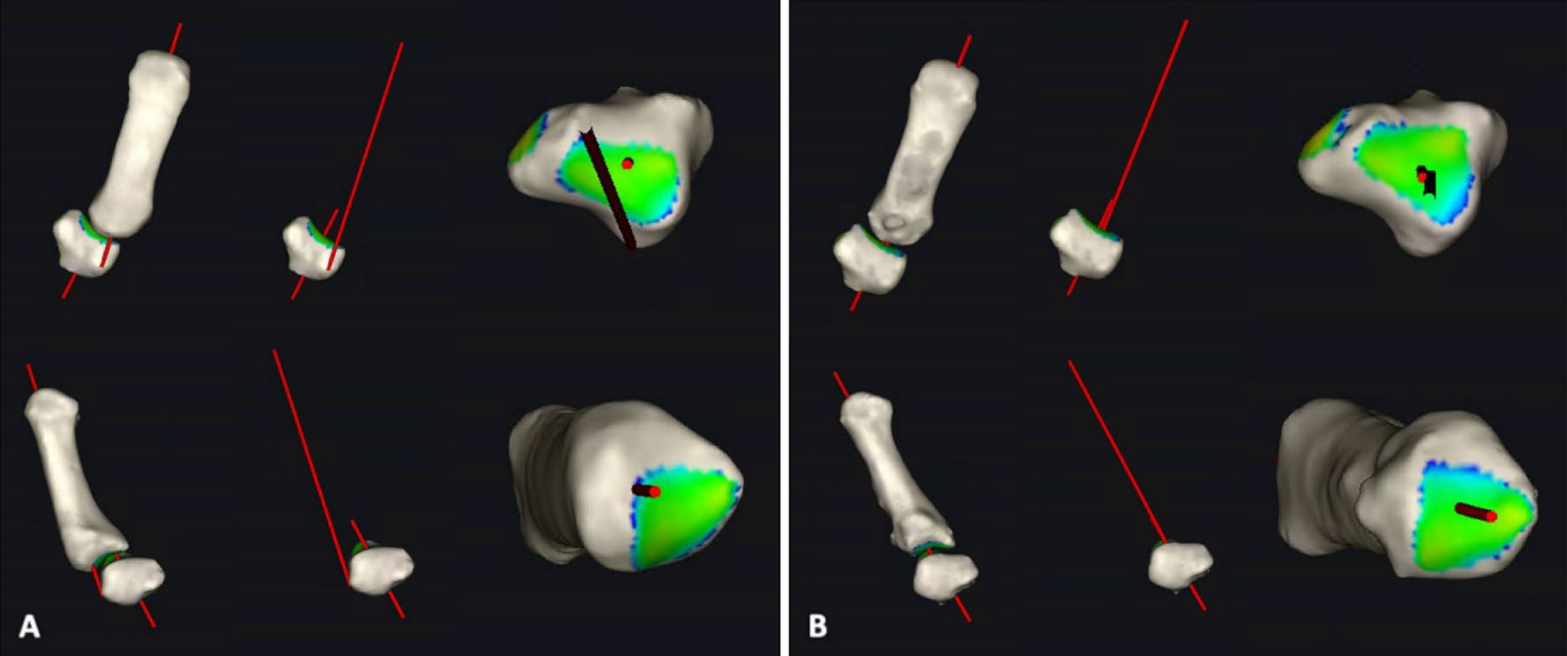
Pre- **A** and postoperative **B** relation between the inertia axes of the MC I and Trapezium (an approximation can also be observed between the inertia axes of these two adjacent bones)

The proximal final point of the mechanical axis is the intersection between the two motion arcs of the proximal biconcave joint surface of the MC I [15]. This corresponds to the highest point of the joint of the base of the MC I. The line between the most proximal radial and ulnar aspect of the proximal MC I joint surface is the proximal joint line (PJL). By approximating the direction of the MA to the one of the AA we believe to aim for an angle of around 90 degrees between the MA/AA and the PJL to sufficiently unload the palmar area of the CMC1- joint surface (Figs. 1, 2).

According to this calculation a correction angle (and therefore osteotomy angle) between 15 and 30° seems more realistic.

### Surgical technique

**Fig. 3:**
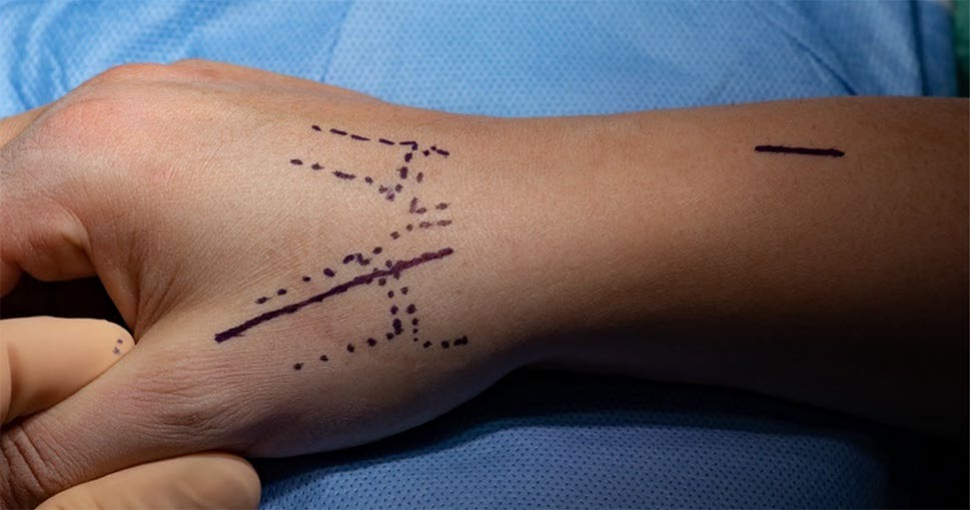
anatomical landmarks and planning of incision

**Fig. 4:**
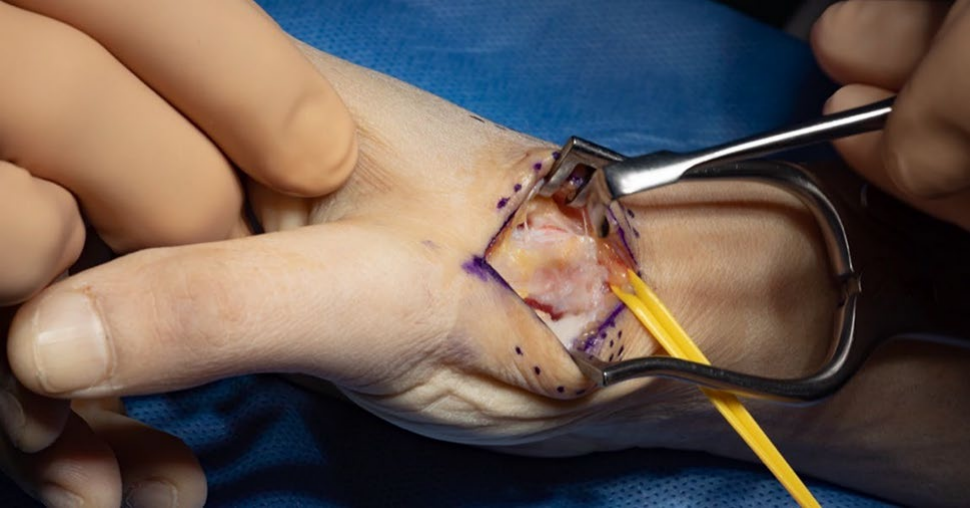
Exposed metacarpal bone and radial artery (yellow loop)

**Fig. 5:**
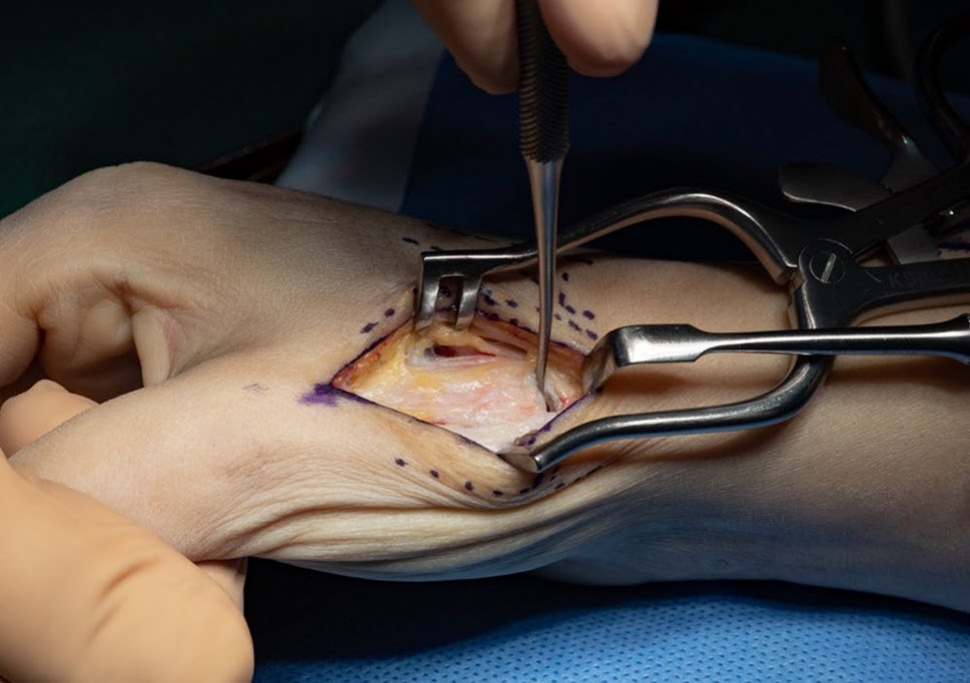
opened CMC I–joint

A dorsal approach to the first carpometacarpal joint is performed, taking care to preserve sensory radial nerve branches (Fig. 3). The radial artery is identified, exposed towards the first web space and marked with a vessel loop (Fig. 4). The periosteum of the first metacarpal is dorsally incised in longitudinal direction and dissected radial- and ulnarwards to expose the bone. The osseus insertion of the APL tendon must be left intact. Horizontal arthrotomy is performed to inspect the cartilage of the joint surfaces and rinse the joint (Fig. 5).

**Fig. 6:**
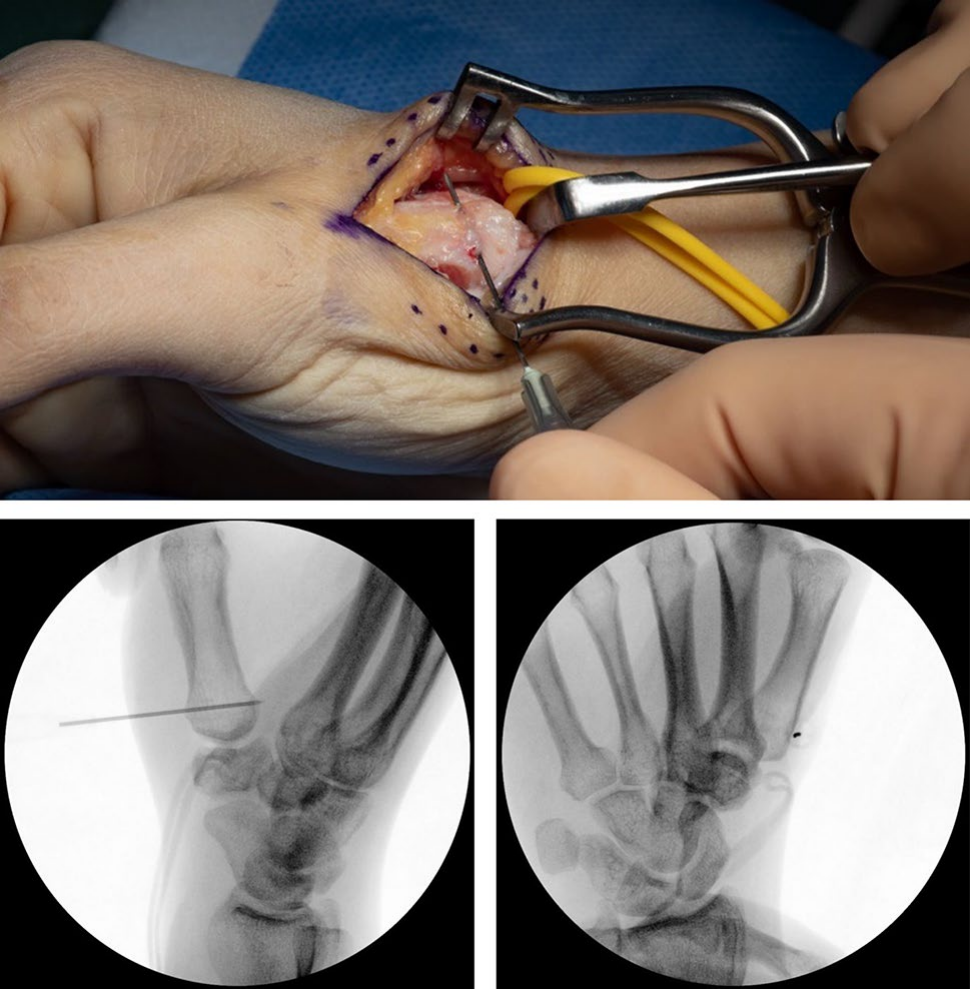
Upper: proximal level of osteotomy (needle), lower: fluoroscopy dorso-palmar and lateral

**Fig. 7:**
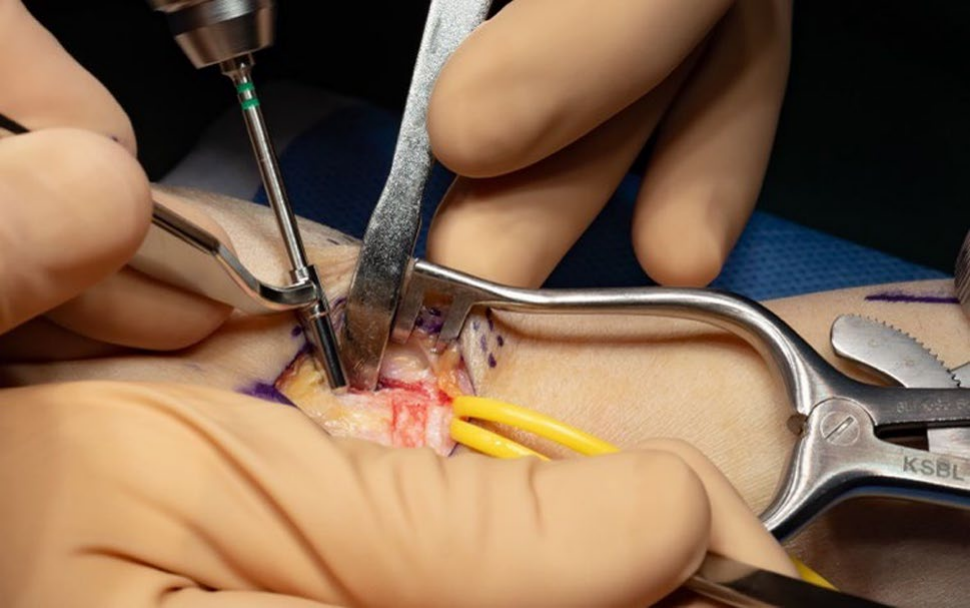
Dorso-ulnar drill hole for reposition clamp

**Fig. 8:**
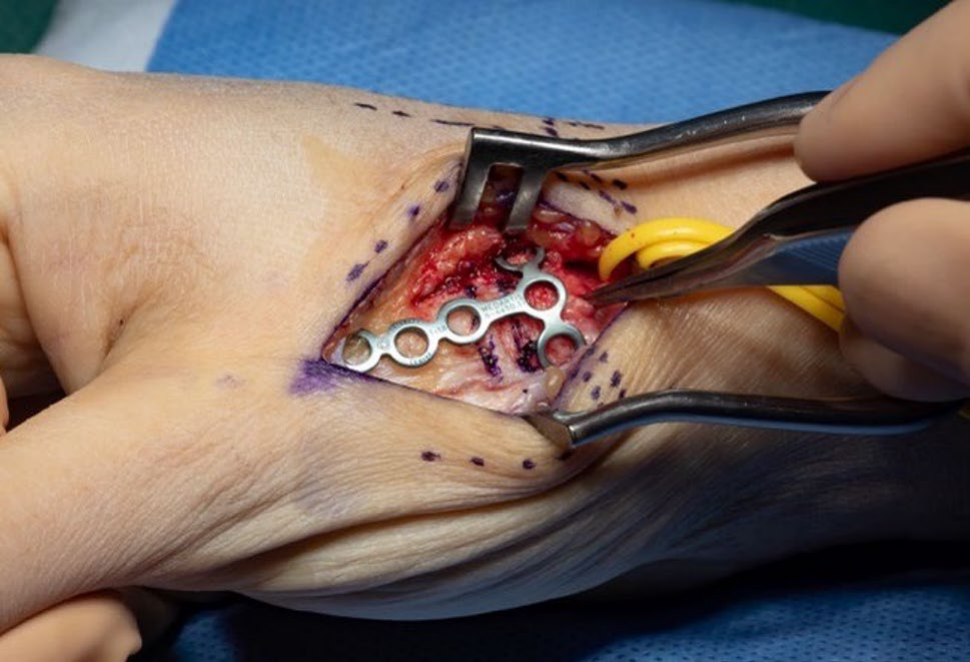
Marked distal and proximal level of osteotomy and future position of the plate and predrilled plate hole in the proximal central plate hole

**Fig. 9:**
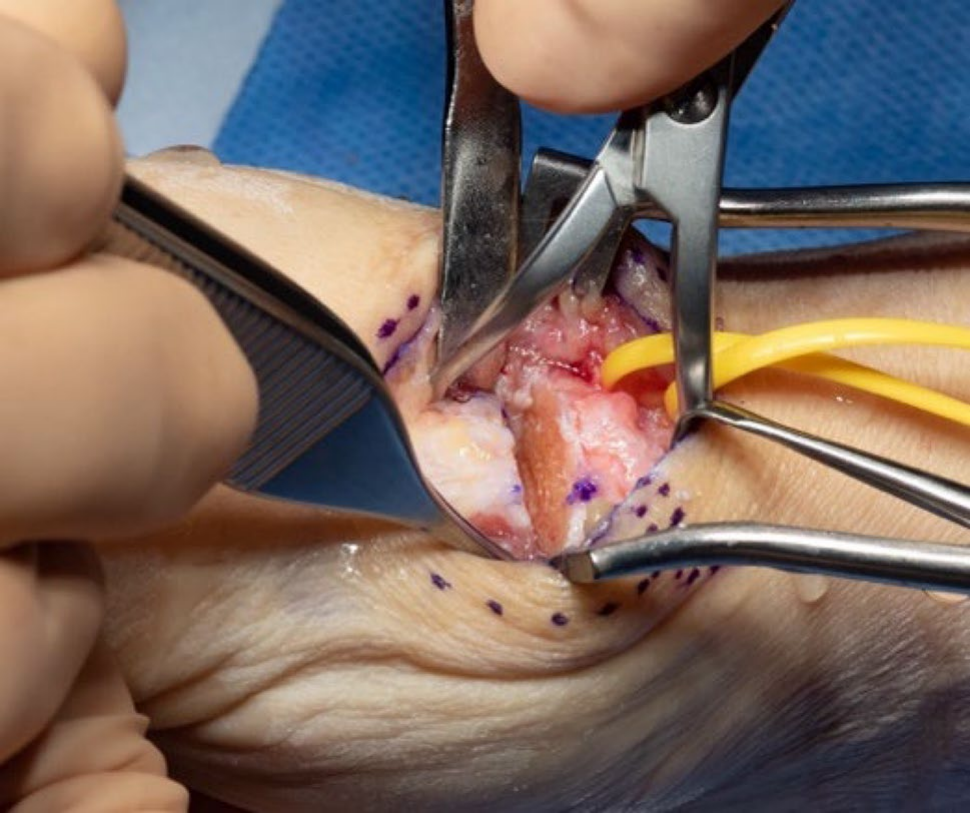
Open osteotomy site with clamp

**Fig. 10:**
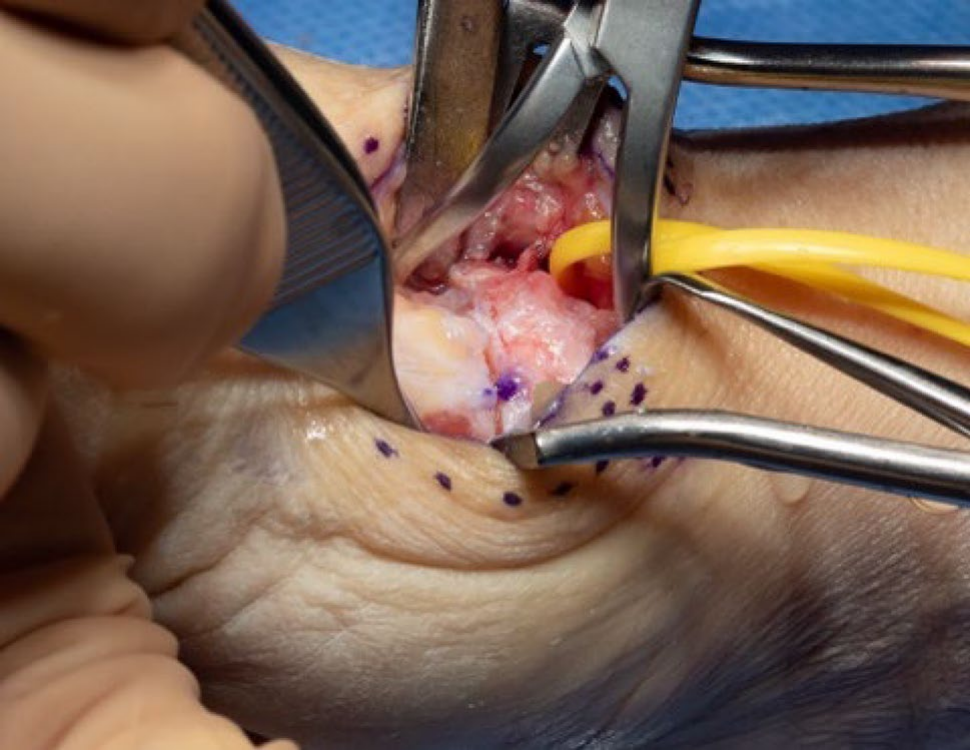
Closed osteotomy

**Fig. 11:**
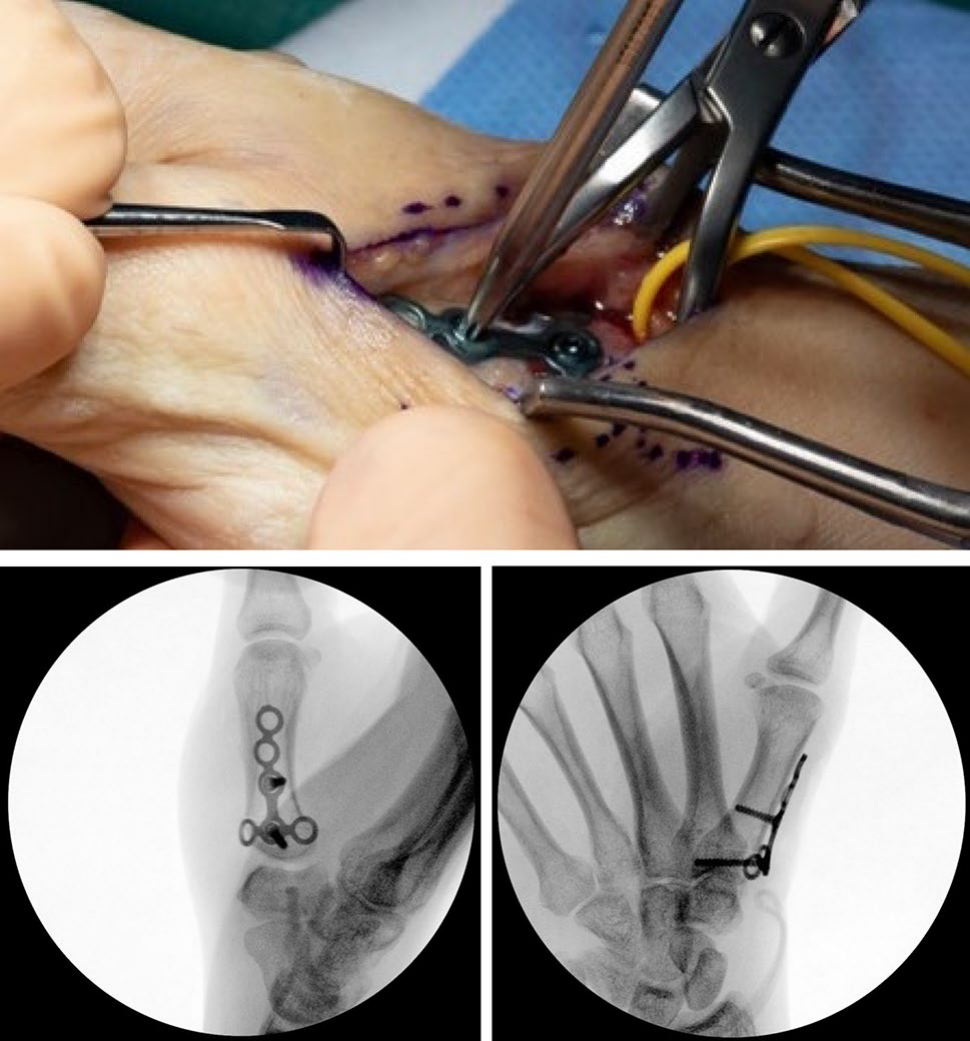
Upper: preliminary fixed plate and compressed osteotomy; lower: fluoroscopy dorso-palmar and lateral

The osteotomy site is chosen under fluoroscopic control with a canula to accommodate ample space for the T-plate (Fig. 6). A centrally placed hole is drilled in the dorsal base of the first metacarpal. It is used for the proximal center plate position. A secondary unicortical drill hole in the dorso-ulnar aspect of the shaft will later facilitate osteotomy reduction (Figs. 7, 8). The osteotomy is performed with an oscillating saw perpendicular to the first metacarpal base, leaving the palmar cortex intact. The second osteotomy is placed about 5 mm more distal, aimed to converge with the proximal osteotomy, again leaving the palmar cortex intact. The dorsal wedge can then be removed, and the osteotomy site is closed slowly with a bone reduction clamp (placed at the base of the MC I and the unicortical dorso-ulnar hole) (Figs. 9, 10). To provide primary stability, we use a T-shaped plate (Medartis AG, Basel, Switzerland) which is pre-bent, to accommodate the dorsally closing wedge osteotomy, usually around 20–30° according to the preoperative planning. The plate is fixed in the proximal central hole and oriented axially. The closest screw hole just distally to the osteotomy is drilled eccentrically to allow for additional compression of the osteotomy site. All remaining holes are then occupied with locking screws (Fig. 11).

**Fig. 12:**
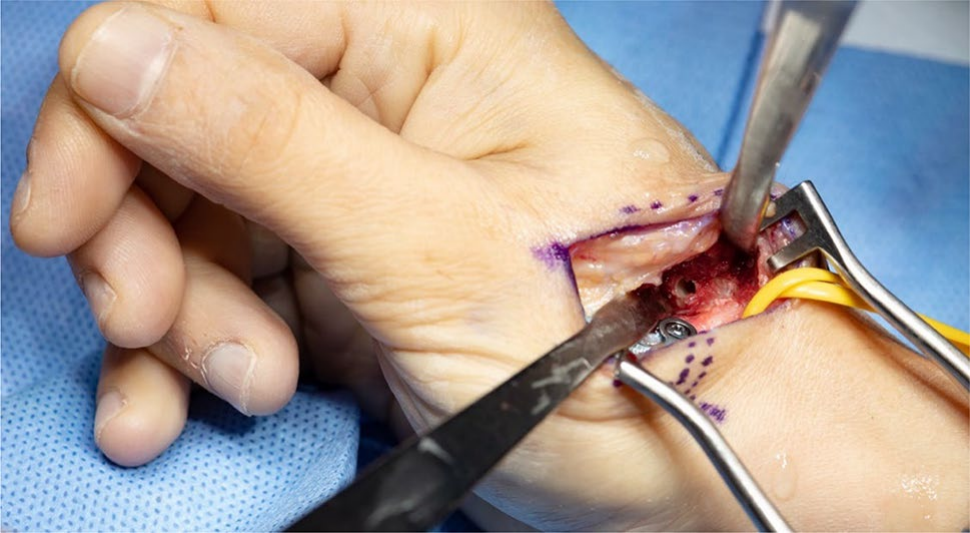
Drill hole of the channel in the base of the metacarpal II

**Fig. 13:**
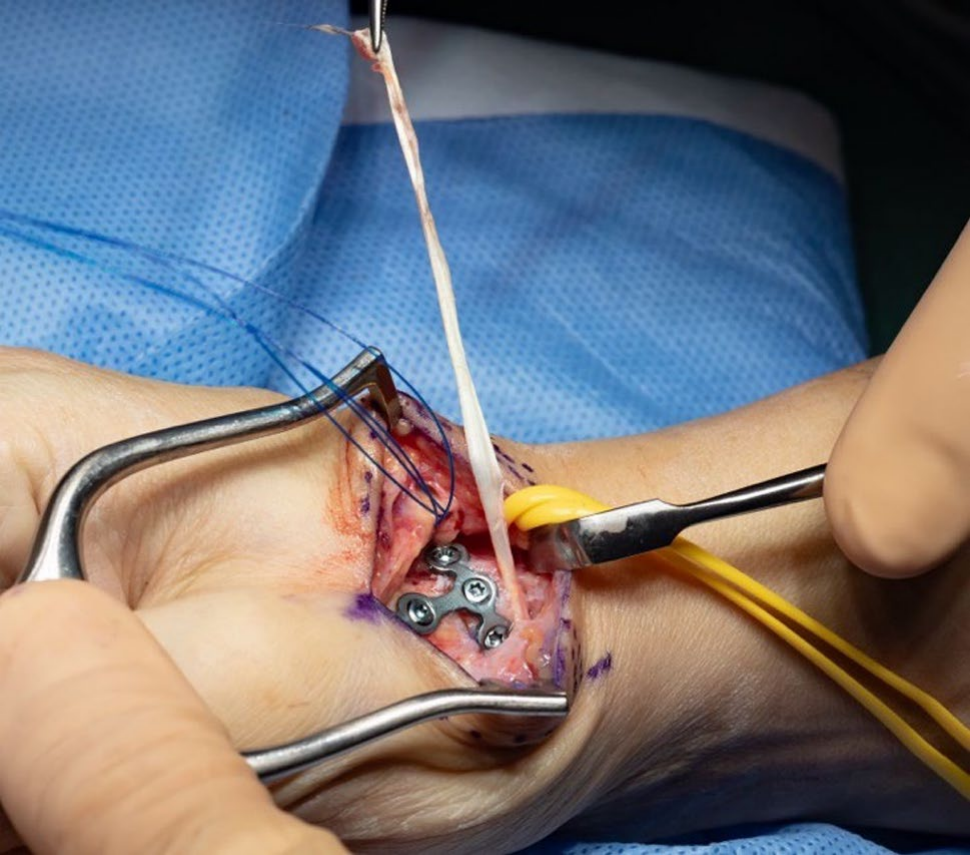
Harvested APL-tendon strip

**Fig. 14:**
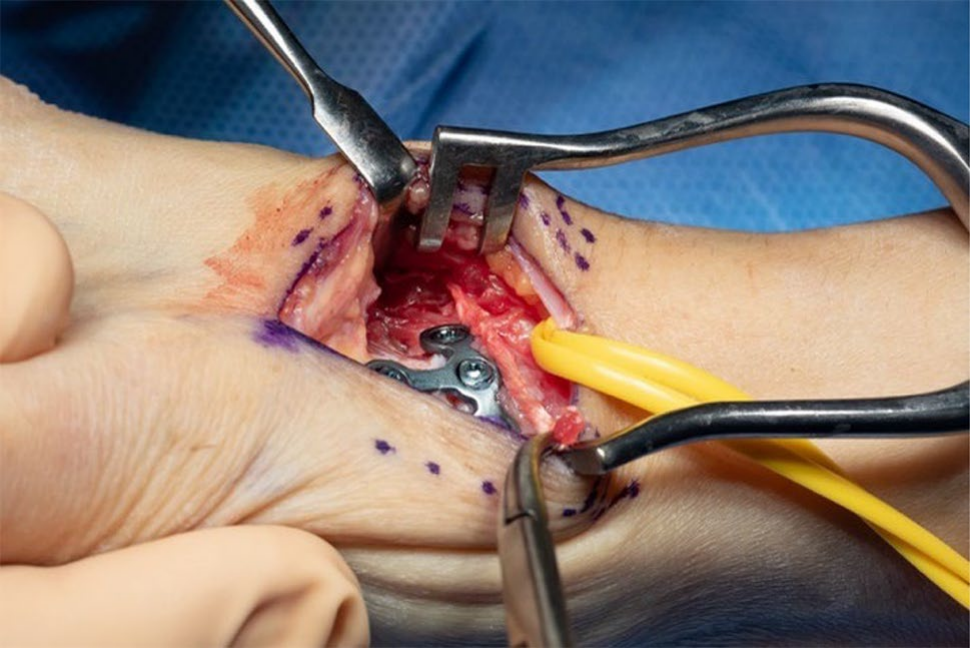
Tightened tendon strip

**Fig. 15:**
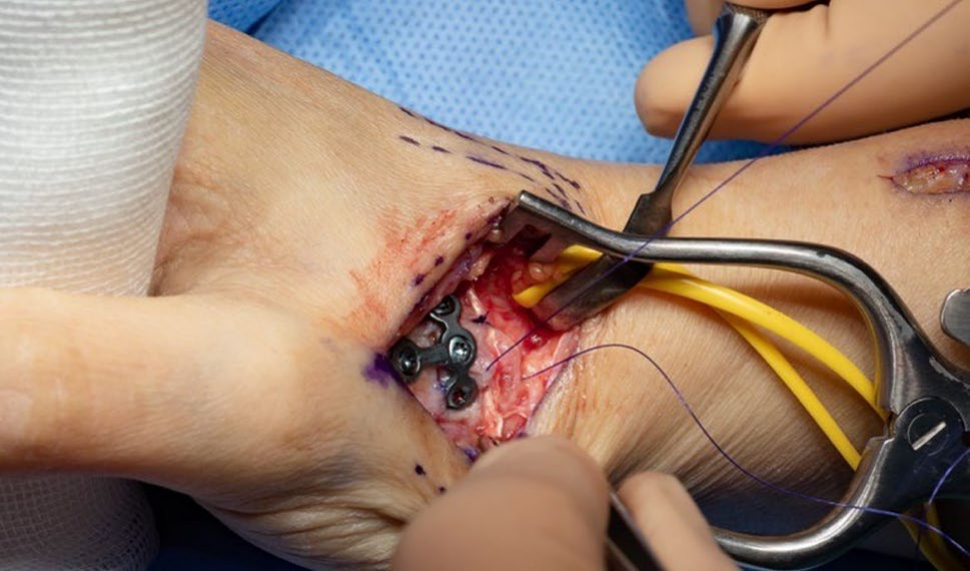
Closure of the joint capsule including the tendon strip

**Fig. 16:**
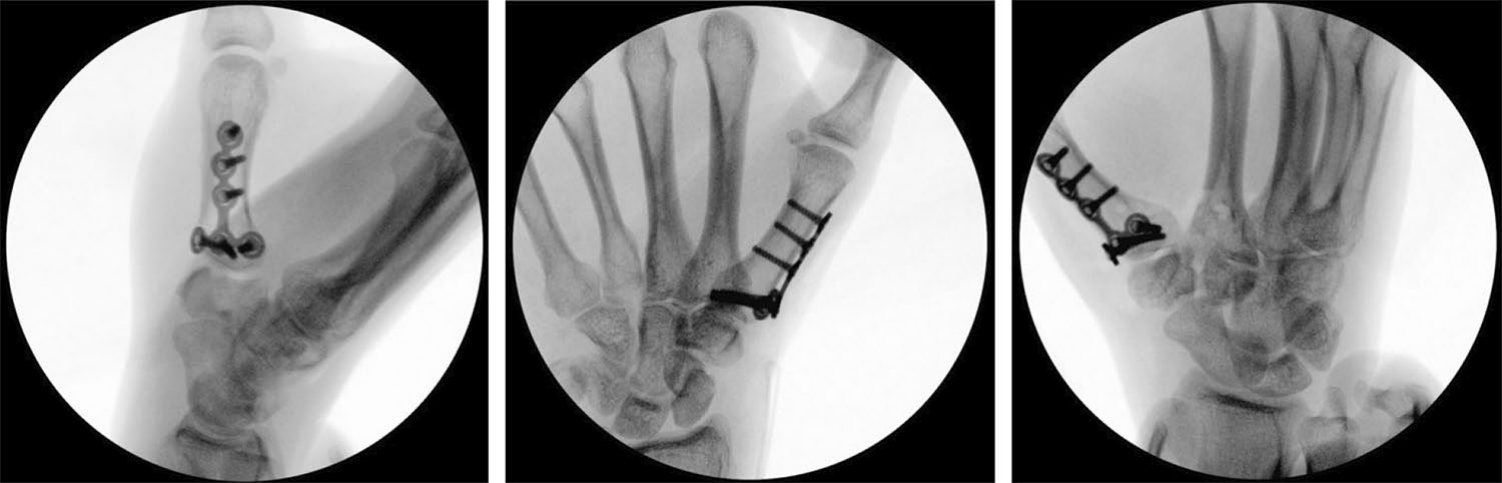
Final intraoperative fluoroscopy

**Fig. 17:**
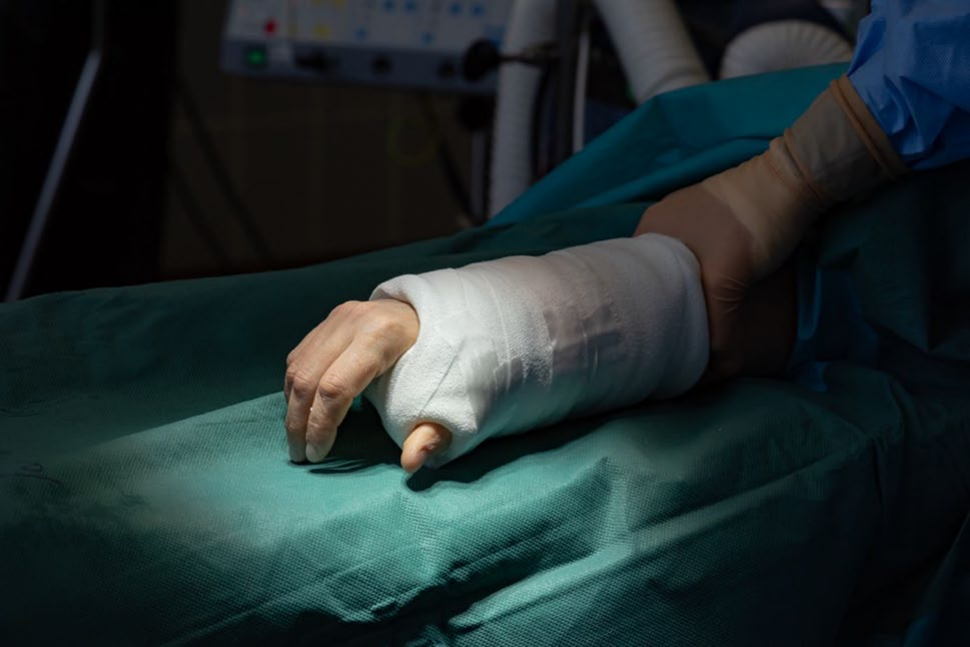
Intraoperatively applied cast

Thereafter, stability of CMC I-joint is assessed. Increased passive motion/subluxation especially in the radio-ulnar direction implies instability [2]. If marked instability persists, a distally based APL tendon slip is harvested usually about one-third to one-half of the dorsal part of the tendon is used. Either via a secondary incision or through the same incision, the radial base of the second metacarpal is exposed. Two converging unicortical holes are drilled in a V shape (> 90°) into the palmar radial and dorsal aspect of the second metacarpal bone (Figs. 12, 13). The APL tendon slip is routed through the CMC I capsule at the level of the base of the MC I from radial to ulnar and then under the radial artery to the base of the second metacarpal. The tendon graft is routed through the drill holes from palmar radial to dorsal, then back towards the CMC I-joint, where it is sutured onto itself with figure-of-eight stitches (Fig. 14). The capsule is closed with a running stitch (Fig. 15). Stability is then reassessed and final documentation using fluoroscopy (Fig. 16). Wound closure is conducted after opening of the tourniquet and confirming integrity of the radial artery. Finally, a comfortable wound dressing and splint is applied (Fig. 17).

**Fig. 18:**
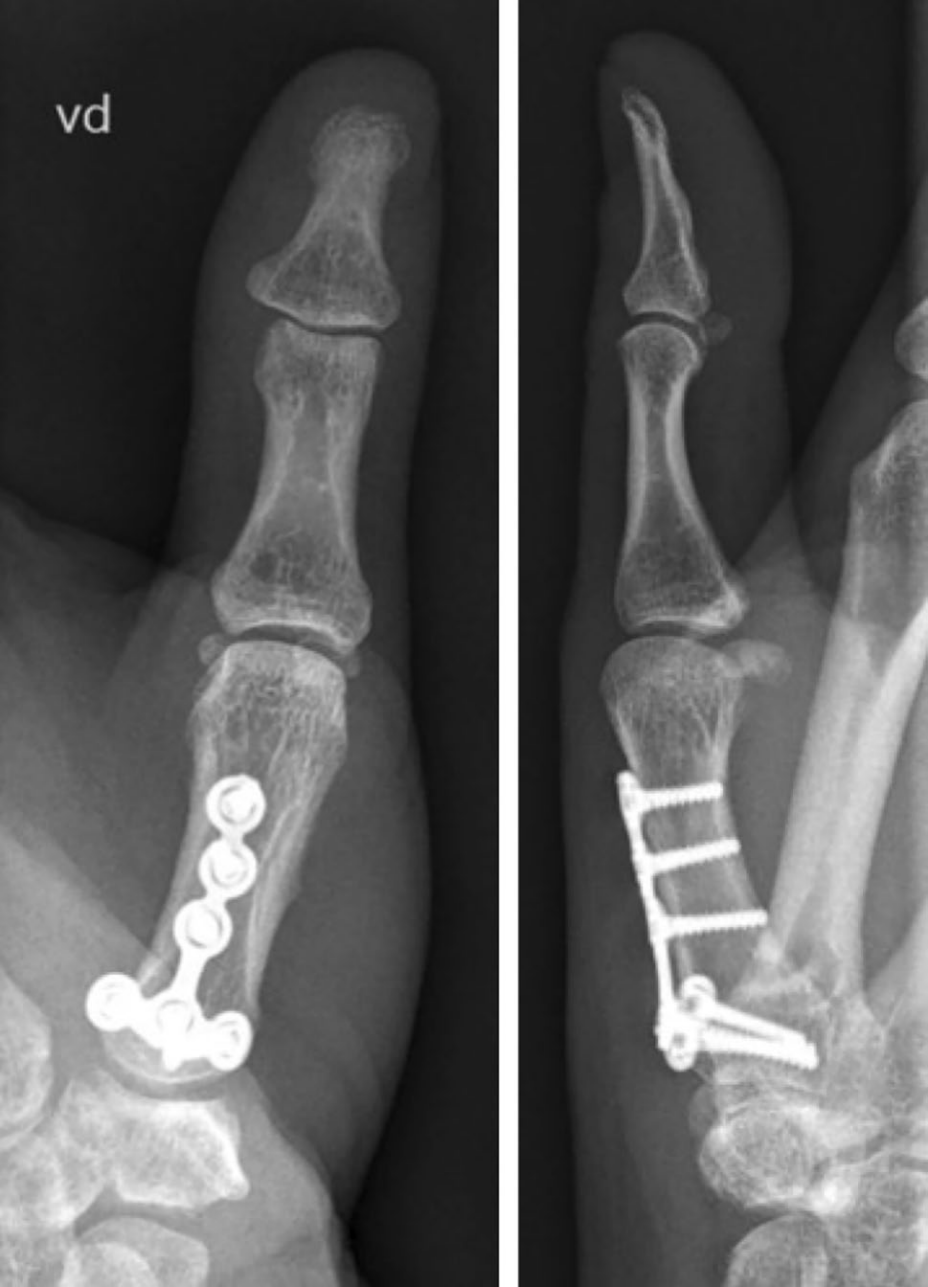
Follow-up 6 weeks postoperatively

Postoperatively, a thumb splint leaving the interphalangeal joint free is applied for 6 weeks with immediate start of early active motion during occupational therapy. Movement with force is started after radiological control at 6 weeks postoperatively and confirmation of bony consolidation (Fig. 18).

## Complications

Beside the common complications related to surgical interventions like infection, wound healing and scar problems, specific procedure related problems are injury of the branches of the superficial radial nerve, tendon irritations, lesion of the radial artery, overcorrection, and non-union. Implant-related problems such as loosening, breakage, and hardware irritation are possible.

Complications related to the ligamentoplasty are rare. If the bone bridge between the unicortical drillholes of the MC II breaks, the APL-slip can be fixed using a Swivelock anchor at the base of the second metacarpal. Care must be taken to guide the tendon slip palmar to the radial artery within the first webspace.

To avoid damage to the radial artery at the level of the anatomical snuffbox, we always identify the radial artery and mark it with a vessel loop.

A 1.5 plate is sufficient to allow stable fixation and primary bone healing. We never encountered a non-union or plate breakage. Many patients consider the proximal end of the plate bothersome which makes a removal of implant necessary.

## Discussion

A few, mostly retrospective, case series have shown positive results of first metacarpal bone extension osteotomy and the procedure is often considered an alternative to ligament reconstruction in early-stage trapeziometacarpal osteoarthritis. Parker reported successful use in eight patients with mild to moderate residual pain in stages I–III trapeziometacarpal osteoarthritis [16]. Bachoura et al. described positive results in 32 thumbs of 28 patients, however, the use of K-wires was responsible for complications in nine cases including two cases of osteomyelitis [17]. The most recent surgical technique has been published by Tomaino et al. in 2011, a K-wire combined with box-wiring were used to perform osteotomy fixation [18].

Badia described an extension osteotomy along with his arthroscopic classification of CMC I-osteoarthritis performing articular debridement and an extension osteotomy with a single K-wire [19]. Joint instability was not addressed. In our opinion, osteosynthesis with a single K-wire does not provide rotational stability and therefore may not allow for early active motion postoperatively.

Chou provided long term results of 13 patients of which 10 had good to excellent results at mean follow-up after 9 years and stated that Wilsons-OT is a good alternative to a ligamentoplasty in patients with early-stage CMC I arthritis [20].

First results of Wilson-OT using patient-specific instrumentation has been described in eight patients by the group of Andreas Schweizer. Their results show high precision of the procedure using the guides [21]. A standardized osteotomy of 20° extension and 5° ulnar adduction has been carried out in all of their patients. They didn’t report on any instabilities of the CMC I-joint in their patient population and did not address this issue during the surgical intervention either.

We perform extension osteotomy in stages I and II. Osteotomy is closed using a T-shaped plate instead of K-wires. Additionally, arthrotomy is routinely performed to assess the stage of osteoarthritis and to rinse out potentially chondro-active metabolites. Stability of the joint is assessed after extension osteotomy. If laxity remains, a modified Brunelli ligament reconstruction is additionally performed. Our short term results in 39 patients (7 male, 32 female; 46 osteotomies) with a follow-up of 11.6 months (range 3–36 months) showed a distribution of 23 CMC I being stage I and 23 being stage II, respectively. Mean age was 45.7 years (range 22–60 years) and an affection of the dominant hand in 79.5%. A postoperative opposition of > 8 was possible in 40 and between 5 and 7 in 6 thumbs. The postoperative key pinch strength was 140% (mean 96%) compared to preoperative. Plate removal was necessary in 22 thumbs.

Since instability of the CMC I-joint and a wrong load bearing axis of the metacarpal are the main contributors to the progress of osteoarthritis, we believe that the corrective osteotomy needs to be combined with a joint-stabilizing ligamentoplasty although the number of plate removal (about 48%) seems to be high due to disturbing osteosynthesis material.

We believe that there seems to less progression of OA because of the correction of the axis and stabilization as well as due to the distraction of the CMC I joint which has been observed previously [22, 23].
