# Correction to: Extension osteotomy of the metacarpal I and ligamentoplasty of the trapeziometacarpal joint for the treatment of early‑stage osteoarthritis and instability of the trapeziometacarpal joint

**DOI:** 10.1007/s00402-023-04942-7

**Published:** 2023-06-23

**Authors:** Philipp Honigmann, Marco Keller, Noémie Devaux‑Voumard, Enrico Coppo, Damian Sutter

**Affiliations:** 1 Hand and Peripheral Nerve Surgery, Department of Orthopaedic Surgery and Traumatology, Kantonsspital Baselland (Bruderholz, Liestal, Laufen), Bruderholz, Switzerland; 2 Medical Additive Manufacturing Research Group (MAM), Department of Biomedical Engineering, University of Basel, Allschwil, Switzerland; 3 Amsterdam UMC, Department of Biomedical Engineering and Physics, University of Amsterdam, Amsterdam Movement Sciences, Amsterdam, The Netherlands; 4 Division of Plastic Surgery and Hand Surgery, University Hospital Zürich, University of Zürich, Zurich, Switzerland; 5 Department of Plastic and Hand Surgery, Inselspital, University Hospital, University of Bern, Bern, Switzerland

**Correction to: Archives of Orthopaedic and Trauma Surgery (2023)** 10.1007/s00402-023-04883-1

The article Extension osteotomy of the metacarpal I and ligamentoplasty of the trapeziometacarpal joint for the treatment of early‑stage osteoarthritis and instability of the trapeziometacarpal joint, written by Philipp Honigmann, Marco Keller, Noémie Devaux‑Voumard, Enrico Coppo and Damian Sutter, was originally published electronically on the publisher’s internet portal on 19 May 2023 without open access. With the author(s)’ decision to opt for Open Choice the copyright of the article changed on 02 June 2023 to © The Author(s) 2021 and this article is licensed under a Creative Commons Attribution 4.0 International License, which permits use, sharing, adaptation, distribution and reproduction in any medium or format, as long as you give appropriate credit to the original author(s) and the source, provide a link to the Creative Commons licence, and indicate if changes were made. The images or other third party material in this article are included in the article’s Creative Commons licence, unless indicated otherwise in a credit line to the material. If material is not included in the article’s Creative Commons licence and your intended use is not permitted by statutory regulation or exceeds the permitted use, you will need to obtain permission directly from the copyright holder. To view a copy of this licence, visit http://creativecommons.org/licenses/by/4.0/.

The original article has been corrected.

